# Corals-inspired magnetic absorbents for fast and efficient removal of microplastics in various water sources†

**DOI:** 10.1039/d4ra02521k

**Published:** 2024-04-15

**Authors:** Yunyan Li, Huilan Chen, Shuai Li, Luping Feng, Ziyi Wang, Di Wang, Qidong Wang, Hua Wang

**Affiliations:** a Huzhou Key Laboratory of Medical and Environmental Application Technologies, School of Life Sciences, Huzhou University Zhejiang 313000 P. R. China Wanghua@zjhu.edu.cn

## Abstract

Microplastics (MPs) as the formidable pollutants with high toxicity and difficult degradation may threaten the aquaculture industry and human health, making it highly necessary to develop the effective removal methods. In this article, Fe_3_O_4_ nanoparticles (NPs) were initially fabricated with mesoporous structure, but showing undesirable adsorption efficiencies for the adsorption of MPs (lower than 70%). Inspired by the reefs-rebuilding corals acting as the sinks for various marine pollutants like plastic, Fe_3_O_4_ NPs were coated further with adhesive polymerized dopamine (PDA) yielding Fe_3_O_4_@PDA absorbents. Unexpectedly, it was discovered that the corals-mimicking absorbents so formed could allow for the removal of MPs with dramatically enhanced efficiencies up to 98.5%, which is over about 30% higher than those of bare Fe_3_O_4_ NPs. Herein, the PDA shells might conduct the increased adhesion to MPs, presumably through the formation of hydrogen bonding, π–π stacking, and hydrophobic interactions. A fast (within 20 min) and stable adsorption of MPs can also be expected, in addition to the PDA-improved environmental storage of Fe_3_O_4_ NPs. Subsequently, the Fe_3_O_4_@PDA adsorbents were utilized to remove MPs from different water sources with high efficiencies, including pure water, suburban streams, village rivers, lake water, inner-city moats, and aquaculture water. Such a magnet-recyclable adsorbent may provide a new way for rapid, effective, and low-cost removal of MPs pollutants from various water systems.

## Abstract 2

Mesoporous Fe_3_O_4_ nanoparticles were coated with polymerized dopamine to mimic corals for adsorption of microplastics from different water sources.

## Graphical abstract

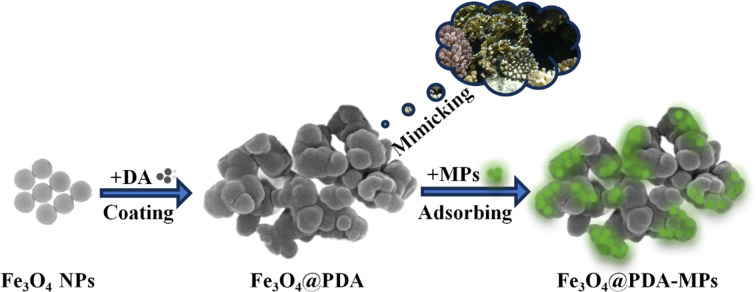

## Introduction

From the beginning of the 20th century, plastic products have come into public life for daily uses, where household, industrial, and fishing activities are the main sources of plastics.^1^ In particular, the outbreak of 2019 coronavirus has triggered a sharp increase in disposable medical and household plastic products, which may increase by 12 billion metric tons by 2050.^2^ Moreover, microplastics (MPs), as a kind of plastic pollutants with higher toxicity, may possess larger specific surface area, making it easier to adsorb various pollutants in the environment.^3^ Also, they can have a serious impact on the aquaculture industry due to their difficult degradation so as to threaten the natural organisms.^4^ When aquatic organisms merely inhale some trace amounts of MPs, they will be accumulated in the aquatic organisms. What is more, it is of current concern that one of the significant sources of MPs ingested by humans may commonly come from the fisheries aquatic products. Once these fishes and shellfishes enter the human body, they may reach the gastrointestinal tracts to be absorbed, with the potential for transfer MPs to the other tissues,^5^ since they may neither be absorbed into the digestive system nor be completely excreted.^6^ For example, MPs may continue to affect the people's immune system, respiratory system, digestive system, and reproductive system, thus endangering the human health.^7^

Currently, the degradation and removal of MPs include physical method,^8^ chemical route,^9^ biological way,^10^ and other combined technologies.^11^ Nevertheless, most of them may encounter with some different limitations in terms of the removal conditions, low efficiencies, and high costs.^12^

Due to magnetic materials can present some advantages of easy preparation, low toxicity, low cost, and magnetic recovery,^13^ they have been extensively used as the functional adsorbents carriers for the adsorption-based removal of various pollutants in the environmental water with the reusable merits.^14^ For example, magnetic adsorbents have been applied for removing some highly-toxic heavy metals ions, such as cadmium,^15^ copper,^16^ chromium,^17^ and mercury^18^ ions. In recent years, some researchers have reported that Fe_3_O_4_ nanoparticles (NPs) might adsorb MPs through the hydrogen bonds and hydrophobic interactions.^19–21^ However, such a magnetic adsorption for MPs might have low capacities and unstable adsorption, which may take too long time (*i.e.*, 150 min) to be practically applied.^19^ What's more, due to the high chemical activity, Fe_3_O_4_ NPs may be easily agglomerated and oxidized in air and/or water, and especially may be corroded in an acidic environment.^22^ Alternatively, increasing studies have been focused on the combination of magnetic materials with other kinds of inorganic and organic adsorption materials for improving the removal efficiencies of various pollutants.^23^ For example, the removal of dyes from water was proposed by using magnetic nanocomposites.^24^ Fe_3_O_4_-loaded zeolitic imidazolate frameworks were also fabricated for removing polystyrene microspheres with a diameter of 1.1 μm.^25^ Moreover, it is well known that dopamine (DA) as a small molecular mimic of adhesion protein from mussels can be readily self-polymerized to form polydopamine (PDA) onto various kinds of inorganic and organic materials by coordination, surface complexation, and electrostatic attraction.^26,27^ Especially, PDA with amine and catechol groups may conduct multiple interface interactions with various pollutants in water such as heavy metals, dyes, and oil.^28–33^

**Scheme 1:**
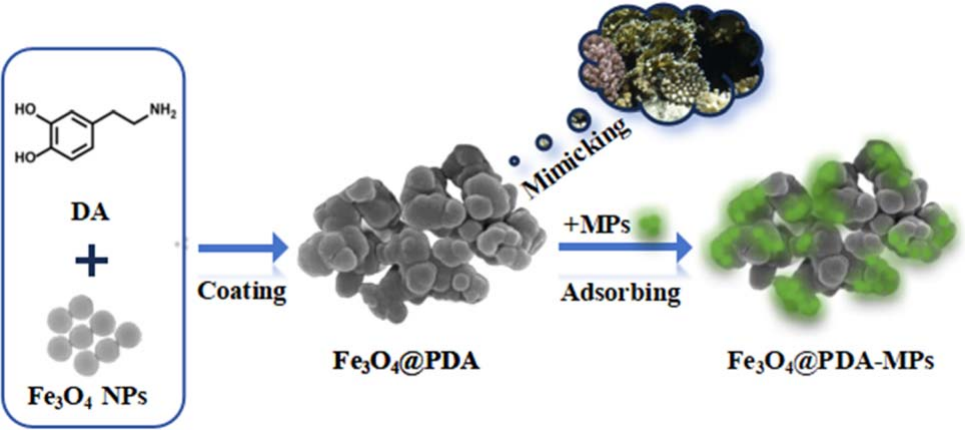
Schematic illustration of mesoporous Fe_3_O_4_ coated with polymerized dopamine to mimic corals for adsorption of MPs.

It has been noteworthy that the coral reefs may naturally serve as the long-term sinks for marine MPs, since they have three-dimensional porous structures and especially some adhesive substances that may actively ingest and concentrate MPs in the sea.^34–36^ In this article, Fe_3_O_4_ NPs were firstly fabricated with mesoporous structure to adsorb MPs, but showing undesirable adsorption efficiencies (lower than 70%). Alternatively, herein, magnetic particles were further coated with adhesive PDA to yield Fe_3_O_4_@PDA nanocomposites by mimicking the natural behavior of corals-building reefs for the removal of MPs. The schematic illustration is shown in Scheme 1. To our surprise, it was discovered that MPs could be adsorbed by the as-prepared nanocomposites with enhanced removal efficiencies up to 98.5%, which is about 30% higher than that of Fe_3_O_4_ NPs alone. In particular, the as-prepared Fe_3_O_4_@PDA could allow for the complete adsorption of MPs within 20 min, in contrast to those of Fe_3_O_4_ NPs that might take much longer time as previously reported.^37^ Especially, PDA so coated could largely enhance the adsorption stability of MPs and environmental robustness of Fe_3_O_4_ NPs, so that Fe_3_O_4_@PDA could basically maintain their adsorption performances without obvious change in harsh environments within 6 months. Moreover, the removing conditions were optimized including adsorbent amounts, pHs, and ion strengths. Subsequently, the developed Fe_3_O_4_@PDA were utilized to remove MPs from different water sources with high removal efficiencies, including pure water, suburban streams, village rivers, lake water, inner-city moats, and aquaculture water, indicating that this magnetic adsorbent may allow for rapid, effective, and low-cost removal of formidable pollutants of MPs from various water sources.

## Materials and methods

### Reagents and apparatus

Ethylene glycol (EG), ferric chloride hexahydrate (FeCl_3_·6H_2_O), sodium acetate (NaAc), hydrochloric acid (HCl), dopamine hydrochloride (DA), sodium hydroxide (NaOH), sodium chloride (NaCl), anhydrous ethanol (C_2_H_6_O), and monodisperse microplastics (MPs) of fluorescent polystyrene (PS) microspheres (1.0 μm, green light) were all purchased from Aladdin (Shanghai, China). No purification was performed, and all solutions were prepared with ultrapure water. Moreover, five kinds of water samples were utilized for testing the removal efficiencies of Fe_3_O_4_@PDA for MPs from different sources of suburban streams, village rivers, lake water, inner-city moats, and aquaculture water, which were collected from the local area of Huzhou City, Zhejiang Province, P. R. China. Fluorescence intensities were recorded by using a fluorescence spectrophotometer (F-7100, Hitachi High-Technologies Corporation, Japan). Transmission electron microscopy (TEM, Tecnai-G20, U.S.A.) and scanning electron microscope (SEM, JSM-6700 F, Japan) were employed to characterize the topological structures of different materials.

### Preparation of Fe_3_O_4_@PDA absorbents

The fabrication of Fe_3_O_4_ NPs was performed by using an improved solvothermal method previously reported.^38^ Typically, an aliquot of FeCl_3_ (3.46 g) was dissolved into ethylene glycol solution (70 mL) by continuously stirring until a clear solution was obtained. Then, NaAc (4.62 g) was added to be stirred for 30 min to obtain a uniform cloudy solution. After that, the mixture was transferred to a 100 mL reactor to be reacted at 200 °C for 8 h. After cooling to room temperature, black magnetic products were obtained through the separation with a permanent magnet. After cleaning with ultrapure water and ethanol, the products were dried in vacuum at 60 °C for 12 h to yield the Fe_3_O_4_ NPs. Furthermore, DA (2.0 mg mL^−1^) and NaOH (2.0 mg mL^−1^) of each 20 mL were fully mixed. Following that, an aliquot of Fe_3_O_4_ NPs (0.20 g) were added to be vigorously stirred at 600 rpm for 30 min at room temperature. Subsequently, magnetic separation, ultrapure water washing, and vacuum drying at 60 °C for 8 h were operated to obtain the Fe_3_O_4_@PDA nanocomposites.

### MPs removal experiments with Fe_3_O_4_@PDA absorbents

An aliquot of Fe_3_O_4_@PDA (20 mg L^−1^) was separately introduced into the solutions containing different concentrations of fluorescent MPs to be stirred at 300 rpm for 60 min at room temperature by using a constant temperature shaker. After the magnetic separation and standing for 60 min, a certain volume (*i.e.*, 200 μL) of the mixtures was taken by a pipette gun at 2.0 cm below the liquid level to measure the fluorescence intensities with a fluorescence spectrophotometer (F-7100, Hitachi High-Technologies Corporation, Japan) at the excitation wavelength of 455 nm and slit width of 10 nm. The concentrations of residual MPs solutions were calculated by the standard curve. Each of the removal experiments was completed in three copies in comparison with the control ones. Herein, the removal efficiencies (RE) of Fe_3_O_4_@PDA on MPs were calculated by using the following equation:
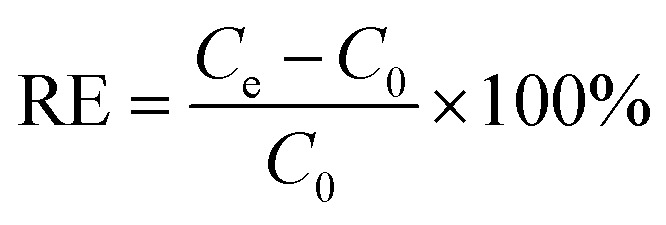
*C*_0_ and *C*_e_ are the mass concentrations of MPs in the solutions before and after the removals, respectively.

Moreover, the removal performances of Fe_3_O_4_@PDA were explored under different adsorption removal conditions, including various pH values (2.0–12), ion strengths in NaCl concentrations (0, 0.0050, 0.010, 0.050, 0.10 M), Fe_3_O_4_@PDA dosages (5.0, 10, 15, 20, 25 mg L^−1^), MPs concentrations (1.0, 2.0, 4.0, 6.0, 8.0 mg L^−1^), temperature (4.0, 10, 25, 50, 80 °C), and adsorption and reaction time. Each of the experiments was performed repeatedly for three times. Subsequently, under the optimal conditions, the Fe_3_O_4_@PDA-based adsorption experiments were performed for different levels of MPs spiked in various water samples, where the MPs samples were prepared separately by mixing five samples that were fetched from various locations of water sources (suburban streams, village rivers, lake water, inner-city moats, and aquaculture water) at time intervals (*i.e.*, 6 h).

## Results and discussion

### Characterization of Fe_3_O_4_@PDA

**Fig. 1:**
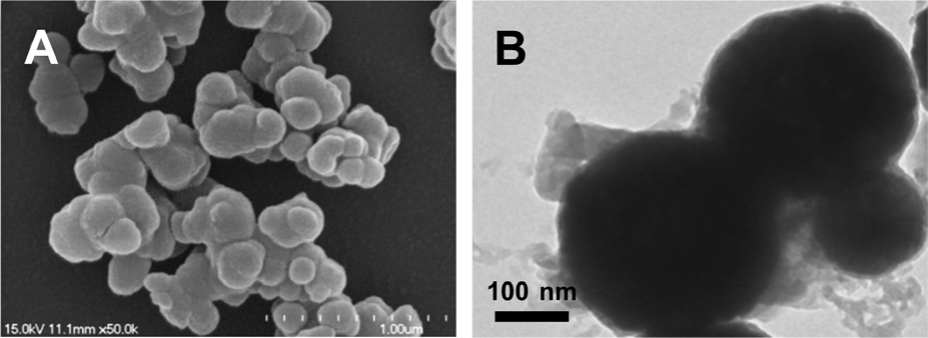
(A) SEM image and (B) TEM image of Fe_3_O_4_@PDA.

Mesoporous Fe_3_O_4_ NPs were fabricated by using the modified solvothermal method,^38^ followed by coating with PDA through the self-polymerized route. Herein, catechol group-containing DA, with chemical structure formula seeing Scheme 1, might conduct strong covalent chelation with Fe_3_O_4_ NPs. The so generated catechol–Fe complex can present the extremely high stability to ensure PDA to be firmly coated onto Fe_3_O_4_@PDA nanocomposites. Fig. 1A explicitly displays that the image of the resulted Fe_3_O_4_@PDA by scanning electronic microscopy (SEM), showing the coral-like shape with the size of about 300 nm in diameter. Especially, PDA coatings might be self-polymerized onto Fe_3_O_4_ NPs in small blocks, of which the adhesive coatings might make Fe_3_O_4_@PDA exist in aggregation way. Furthermore, as revealed in the transmission electron microscopy (TEM) image (Fig. 1B), the resulting Fe_3_O_4_@PDA might present the mesoporous structure to ensure a large specific surface area for loading PDA.

### The adsorption performances of Fe_3_O_4_@PDA for MPs removal

**Fig. 2:**
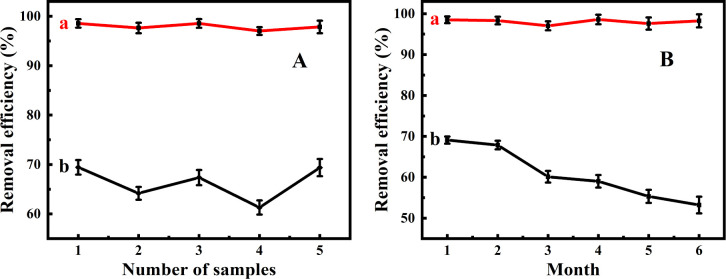
Comparison of (A) adsorption efficiencies and (B) storage stability between (a) Fe_3_O_4_@PDA and (b) Fe_3_O_4_ absorbents of 20 mg L^−1^ for 4.0 mg L^−1^ MPs.

The adsorption performances of Fe_3_O_4_@PDA for MPs removal were initially investigated in comparison Fe_3_O_4_ NPs, where the removal efficiencies were quantitatively determined by measuring the intensities of targeting fluorescent MPs (Fig. 2). It was discovered from Fig. 2A that the removal efficiencies of Fe_3_O_4_@PDA for MPs were achieved over 97.0–98.5%, in contrast to those of Fe_3_O_4_ NPs obtained over the range of 61.3–69.4% at the same dosage (*i.e.*, 20 mg L^−1^). On the one hand, MPs could be adsorbed and aggregated onto the porous Fe_3_O_4_@PDA as coral reefs. On the other hand, the adhesive PDA coatings of the adsorbents should possess a large number of active groups (*i.e.*, amino and catechol groups, seeing Scheme 1) and benzene rings, so that they might display the large adsorption capacities for MPs through the formation of hydrogen bonding, π–π conjugation, and hydrophobic interactions. Herein, the PDA shells with benzene rings might conduct the π–π interaction with the PS MPs with benzene rings. Also, the PDA coatings derivatized with catechol and amino groups might bind with MPs by forming hydrogen bonds to endure the increased adhesion.^39,40^ Moreover, it is worth noting that the hydrophobic interaction between Fe_3_O_4_@PDA and MPs may additionally affect the adsorption efficiencies of MPs. In addition, Fe_3_O_4_ carriers might endow the Fe_3_O_4_@PDA with the magnetic separation performance, making them be recycled and reused with the assistance of magnets. Yet, the detailed mechanism for the improved adsorption of Fe_3_O_4_@PDA for MPs should be studied further. Moreover, one can find from Fig. 2B that Fe_3_O_4_@PDA could exhibit the MPs-adsorption capacities unchanged within six months of storage, whereas Fe_3_O_4_ NPs might display the adsorption capacities that rapidly decreased after the storage beyond two months. Therefore, the PDA coatings should additionally enhance the environmental stability of Fe_3_O_4_ carriers during storage time.

**Fig. 3:**
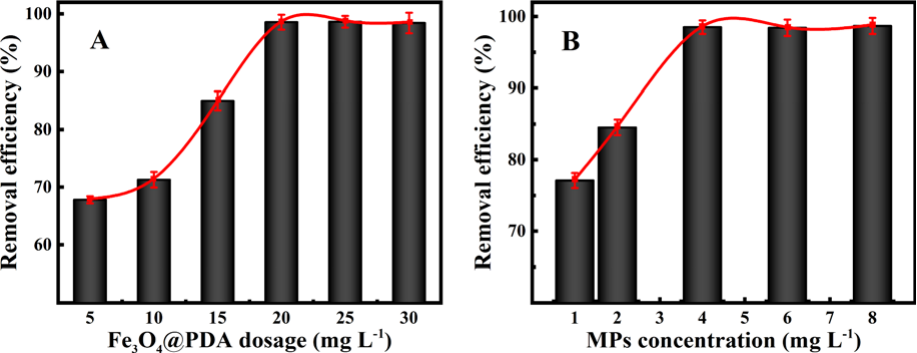
Effects of various (A) Fe_3_O_4_@PDA absorbents of 4.0 mg L^−1^ MPs and (B) different MPs concentrations on the removal efficiencies at 20 mg L^−1^ Fe_3_O_4_@PDA.

The experimental data provided in Fig. 3A show that the removal efficiencies of MPs can significantly increase with the increasing Fe_3_O_4_@PDA dosages. One can note that the removal efficiencies of MPs shift from 67.8% to 98.5% by using Fe_3_O_4_@PDA in the amount range of 5.0–20 mg L^−1^, over which the adsorption efficiencies might tend to be stable. Furthermore, in order to better understand the adsorption characteristics of MPs, different initial concentrations of MPs were applied for the analysis of the adsorption isotherm model. As shown in Fig. 3B, the removal efficiencies of MPs are significantly larger at the high initial concentrations than at the low initial ones using the same amount of Fe_3_O_4_@PDA. Accordingly, the removal efficiencies can maintain around 98.5% for MPs in the range of 4.0–10 mg L^−1^. Such a phenomenon might presumably be resulted from the increasing collisions and adsorption sites for the increasing MPs concentrations. Therefore, 20 mg L^−1^ Fe_3_O_4_@PDA was selected as the optimal one in experiments afterwards.

**Fig. 4:**
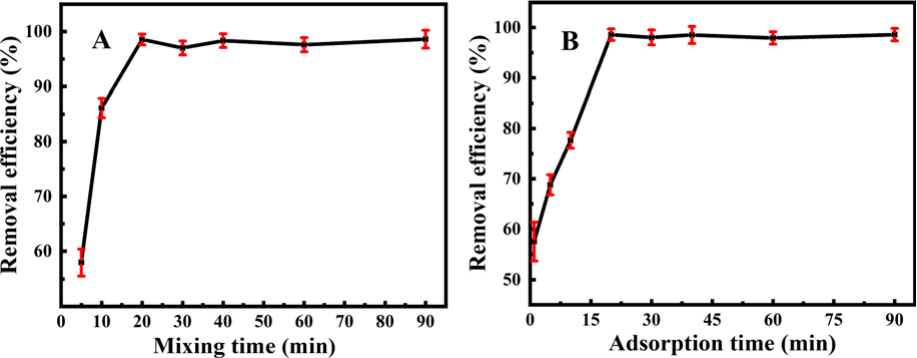
Effects of (A) mixing time and (B) adsorption time on the removal efficiencies of MPs (4.0 mg L^−1^) using 20 mg L^−1^ Fe_3_O_4_@PDA.

One can discover from Fig. 4A that the longer the mixing time of Fe_3_O_4_@PDA with MPs in a constant temperature shaker, the higher the adsorption efficiencies were achieved. As the adsorption tended to be stable after 20 min, the adsorption efficiencies could reach up to 98.5%. Fig. 4B manifests the magnetization treatment time. It was found that with the increase in magnetization time, Fe_3_O_4_@PDA could present the increasing adsorption efficiencies. In the first 10 min, herein, the adsorption efficiencies increased slowly from 57.4% to 77.7%. Interestingly, they might increase sharply from 77.5% to 98.5% over 10–20 min, after which the adsorption efficiencies might gradually go down. Accordingly, the optimal time both for Fe_3_O_4_@PDA-MPs mixing and MPs adsorption are obtained as each of 20 min.

### Effects of adsorption conditions on the removal of MPs

**Fig. 5:**
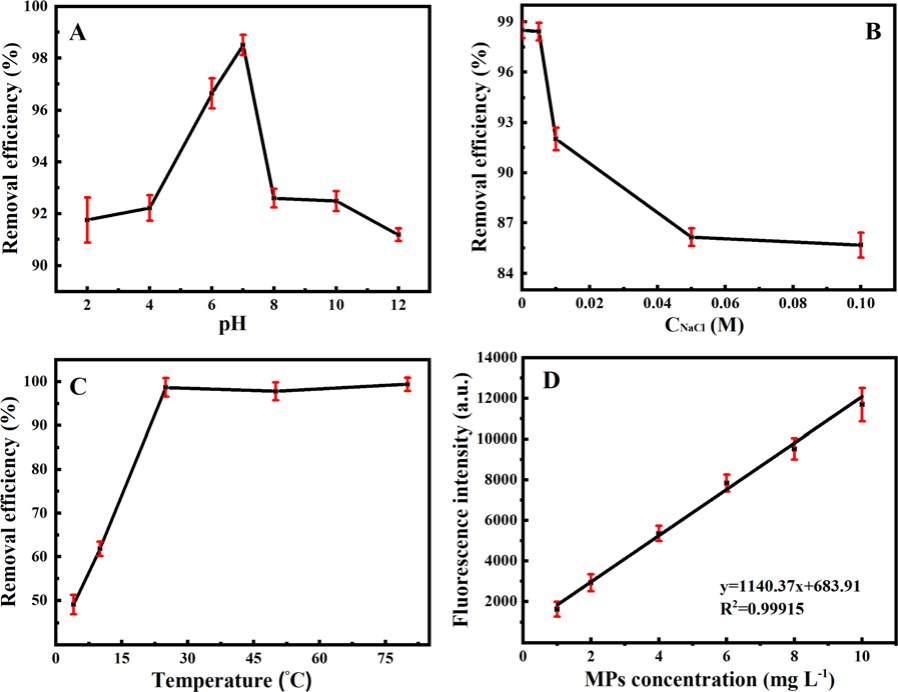
Effects of initial (A) pH values, (B) ion strengths in NaCl concentrations, and (C) temperature on the MPs (4.0 mg L^−1^) removal efficiencies by using 20 mg L^−1^ Fe_3_O_4_@PDA. (D) The standard curve of MPs concentrations.

The adsorption conditions on the removal of MPs were investigated including pH values, ion strengths and temperature (Fig. 5). Fig. 5A manifests that the removal efficiencies of MPs by Fe_3_O_4_@PDA depend on different initial pH values. It is noted that the removal efficiencies of MPs gradually increase with the increase of the initial pH values. At pH 7.0, the removal efficiencies can reach the highest one of 98.5%. When the pH values increased from 7.0 to 12, however, the removal efficiencies of MPs decreased down to 91.2%, which might be resulted from the influence of electrostatic interaction.^37^ Herein, since the PDA coatings possess some active groups (*i.e.*, amino and catechol groups), Fe_3_O_4_@PDA might be positively charged in lower pH range (*i.e.*, pH 2.0) and negatively charged in higher pH range (*i.e.*, pH 12), so as to display the different adsorption capacities for the negatively-charged MPs. Notably, the experimental results indicate that the difference in the removal efficiencies of MPs is not higher than 10%, regardless of the MPs adsorption in acidic, neutral, and alkaline environments, showing no significant effect in general. Moreover, the effects of ion strengths on the MPs adsorption were investigated by using various NaCl concentrations (Fig. 5B). A difference of the removal efficiencies of MPs was observed for changing ion strengths, where the bigger ones were attained at the lower ion strengths. Accordingly, Fe_3_O_4_@PDA might not be applied for the adsorption of MPs in salty media like seawater. Besides, as shown in Fig. 5C, the removal efficiencies of MPs were obtained from 49.0% to 98.5% over the temperature range of 4–25 °C. Higher temperature, interestingly, might enhance the adsorption efficiencies (*i.e.*, 99.4% at 80 °C), presumably due to that the high temperature might accelerate the interactions among Fe_3_O_4_@PDA and MPs. Nevertheless, considering the practical feasibility and energy saving, the optimal temperature for MPs adsorption was subsequently chosen as the room temperature of 25 °C thereafter.

### Fe_3_O_4_@PDA-based removal of MPs in water samples

**Fig. 6:**
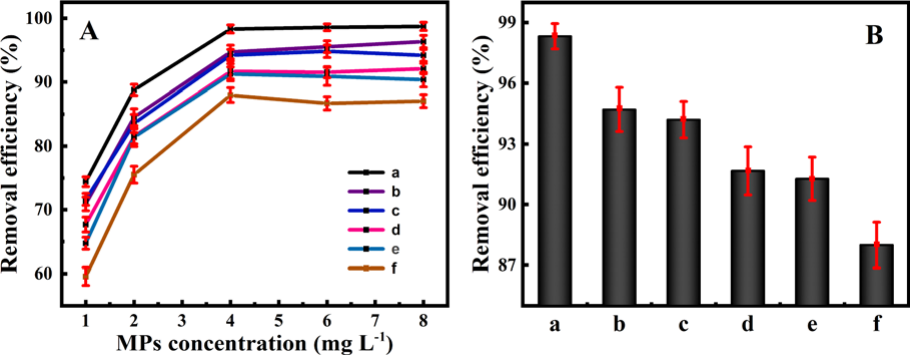
Removal efficiencies of Fe_3_O_4_@PDA (20 mg L^−1^) for MPs of (A) different initial concentrations and (B) 4.0 mg L^−1^ spiked in water samples from different sources of (a) pure water, (b) suburban streams, (c) village rivers, (d) lake water, (e) aquaculture water, and (f) inner-city moats.

Under the optimized adsorption conditions, the linear diagram of MPs concentrations *versus* fluorescence intensities was described for MPs of different concentrations (Fig. 5D). One can see that the fluorescence intensities were almost not detected after magnetic separation without MPs, indicating that Fe_3_O_4_@PDA should exert no influence on the determination of fluorescence intensities of MPs, which might reflect the removal efficiencies of MPs. More importantly, it is noted from Fig. 5D that a linear relationship can be obtained for the evaluation of the adsorption removal of MPs by using Fe_3_O_4_@PDA. Furthermore, the Fe_3_O_4_@PDA-based adsorption strategy was applied for removal of MPs samples from different water sources, including suburban streams, village rivers, Lake water, aquaculture water, and inner-city moats. The results are shown in Fig. 6, of which the raw data are provided in the ESI.†Fig. 6A manifests that the developed Fe_3_O_4_@PDA adsorbents can exhibit the different removal efficiencies for MPs at various concentrations spiked in varying water samples, as shown more clearly for MPs at the same level (Fig. 6B). Accordingly, the removal efficiencies are presented in order: suburban streams > village rivers > lake water > aquaculture water > inner-city moats. It was found that the adsorbents might present higher removal efficiencies of MPs in the less polluted water sources like suburban streams. In contrast, the adsorption of Fe_3_O_4_@PDA for MPs might be interfered by some co-existing substances (*i.e.*, algae, metal ions, and dyes) in the heavily polluted sources like the city center moats, showing the lower removal efficiencies. In general, the experimental data indicate that the adsorption efficiencies of Fe_3_O_4_@PDA for the removal of MPs from the different water sources can be achieved over the range of 62.6–98.5%, demonstrating the feasibility of wide applications for the removal of MPs in different water systems, especially the aquaculture ones.

## Conclusions

To summarize, by mimicking the corals reef’ behaviour in sea, a recyclable absorbent has been successfully fabricated simply by coating adhesive PDA onto mesoporous magnetic carriers for the removal of MPs from different water sources. As compared with the common adsorbents especially bare Fe_3_O_4_ ones, the corals-like Fe_3_O_4_@PDA can present some advantages for MPs removal. First of all, they may enjoy the removal of MPs with high efficiencies (up to 98.5%), which is about 30% higher than that of bare Fe_3_O_4_ NPs (lower than 70%). Moreover, the PDA shells can endow the adsorbents with the larger and especially more stable adsorption of MPs through the formation of hydrogen bonding, π–π stacking, and hydrophobic interactions, which might help to avoid any the second pollution of the formidable targets. Also, the enhanced environmental storage of magnetic adsorbents can be expected, so that the Fe_3_O_4_@PDA can maintain the adsorption capacities without obvious change in harsh environments within six months. In addition, the Fe_3_O_4_@PDA can allow for the magnet-aided recycling adsorption of MPs from a variety of water sources (pure water, suburban streams, village rivers, lake water, inner-city moats, and aquaculture water). Therefore, the developed corals-like adsorbent may promise the wide applications for rapid, effective, low-cost, and recyclable removal of MPs contaminants from various water systems and aquatic products.

## Author contributions

Yunyan Li: data curation, formal analysis, methodology, validation, writing – original draft. Huilan Chen: data curation, software, investigation. Shuai Li: visualization, resources. Luping Feng: formal analysis, investigation. Ziyi Wang, Di Wang, Qidong Wang: investigation, software. Hua Wang: conceptualization, funding acquisition, project administration, supervision, writing – review & editing.

## Conflicts of interest

There are no conflicts to declare.

## Supplementary Material

RA-014-D4RA02521K-s001
