# Supplementary material for: Corals-inspired magnetic absorbents for fast and efficient removal of microplastics in various water sources
Source: RSC Adv. 2024 Apr 15;14(17):11908–13. doi: 10.1039/d4ra02521k (PMC11017187; doi:10.1039/d4ra02521k)
Supplement: RA-014-D4RA02521K-s001 [file RA-014-D4RA02521K-s001.pdf]

Electronic Supplementary Material (ESI) for RSC Advances.
This journal is © The Royal Society of Chemistry 2024

Supplementary Materials

Corals-inspired magnetic absorbents for fast and efficient removal of microplastics in various water sources

Yunyan Li, Huilan Chen, Shuai Li, Luping Feng, Ziyi Wang, Di Wang, Qidong Wang, and Hua Wang*

Huzhou Key Laboratory of Medical and Environmental Application Technologies, School of Life Sciences, Huzhou University, Zhejiang 313000, P.R. China.

* Corresponding Author: E-mail address: wanghua@zjhu.edu.cn; Web: http://wang.qfnu.edu.cn.

Experimental Section

An aliquot of Fe_3O_4@PDA (20 mg L^{-1}) was separately introduced into the solutions of different water sources containing fluorescent MPs of different concentrations, which were stirred at 300 rpm for 20 min at room temperature by using a constant temperature shaker. After the magnetic separation and standing for 20 min, a certain volume (i.e., 200 μL) of the mixtures at 2 cm below the liquid level was taken out by using a pipette gun, of which the fluorescence intensities were measured with a fluorescence spectrophotometer (F-7100, Hitachi High-Technologies Corporation, Japan) at the excitation wavelength of 455 nm and slit width of 10 nm. Moreover, each of the MPs samples from different water sources was prepared separately by mixing five samples that were fetched in different time and/or locations. Accordingly, the removal efficiencies (RE_X) of Fe_3O_4@PDA for MPs were calculated by using the following equation:

$$\Delta F_X = F_0 - F_x \qquad (1)$$

$$RE_X = \frac{\Delta F_X}{F_0} \times 100\% \qquad (2)$$

Herein, F_0 and F_e are the fluorescence intensities of MPs in the solutions before and after the removal operations, respectively, where ΔF_X refer to the differences between their fluorescence intensities.

The raw data for Figure 6 are provided below.

1. Pure water samples

MPs concentrations	F_0	F_X	ΔF_X	RE_X (%)	Average RE (%)	STDEV
1.0 mg L^{-1}	1659.71	412.53	1247.18	75.14	74.42	0.007708
	1646.23	434.46	1211.77	73.61		
	1661.83	423.87	1237.96	74.49		
2.0 mg L^{-1}	2943.87	356.29	2587.58	87.90	88.79	0.008773
	2929.56	327.81	2601.75	88.81		
	2931.12	303.33	2627.79	89.65		
4.0 mg L^{-1}	5474.98	55.76	5419.22	98.98	98.32	0.006261
	5401.99	122.33	5279.66	97.74		
	5468.35	95.61	5372.74	98.25		
6.0 mg L^{-1}	7851.56	71.45	7780.11	99.09	98.58	0.005285
	7842.29	108.43	7733.86	98.62		
	7813.64	153.54	7660.10	98.03		
8.0 mg L^{-1}	9592.56	63.89	9528.67	99.33	98.73	0.006473
	9581.92	115.08	9466.84	98.80		
	9546.48	186.58	9359.90	98.05		

2. Suburban streams samples

MPs concentrations	F_0	Fx	ΔFx	RE_X (%)	Average RE (%)	STDEV
1.0 mg L^{-1}	1883.43	555.61	1327.82	70.50	70.91	0.010764
	1838.93	549.84	1289.09	70.10		
	1801.35	502.00	1299.35	72.13		
2.0 mg L^{-1}	3210.87	529.15	2681.72	83.52	84.53	0.013098
	3284.73	523.59	2761.14	84.06		
	3259.06	455.94	2803.12	86.01		
4.0 mg L^{-1}	5464.27	309.28	5154.99	94.34	94.71	0.010835
	5442.53	221.51	5221.02	95.93		
	5431.89	333.52	5098.37	93.86		
6.0 mg L^{-1}	7912.98	394.86	7518.12	95.01	95.54	0.009354
	7958.65	269.00	7689.65	96.62		
	7943.65	397.98	7545.67	94.99		
8.0 mg L^{-1}	9613.98	285.54	9328.44	97.03	96.35	0.009694
	9599.33	309.10	9290.23	96.78		
	9641.42	458.93	9182.49	95.24		

3. Village rivers samples

MPs concentrations	F_0	F_X	ΔF_X	RE_X (%)	Average RE (%)	STDEV
1.0 mg L^{-1}	1798.12	491.97	1306.15	72.64	71.63	0.009478
	1790.76	523.62	1267.14	70.76		
	1754.85	500.31	1254.54	71.49		
2.0 mg L^{-1}	3014.56	472.68	2541.88	84.32	83.55	0.014212
	3091.38	559.23	2532.15	81.91		
	3054.66	475.92	2578.74	84.42		
4.0 mg L^{-1}	5342.66	298.12	5044.54	94.42	94.21	0.009035
	5391.03	270.09	5120.94	94.99		
	5358.82	363.33	4995.49	93.22		
6.0 mg L^{-1}	8012.32	324.50	7687.82	95.95	94.83	0.009744
	8019.98	455.53	7564.45	94.32		
	8036.55	465.32	7571.23	94.21		
8.0 mg L^{-1}	9780.21	535.96	9244.25	94.52	94.18	0.009469
	9700.54	493.76	9206.78	94.91		
	9776.54	673.60	9102.94	93.11		

4. Lake water samples

MPs concentrations	F_0	F_X	ΔF_X	RE_X (%)	Average RE (%)	STDEV
1.0 mg L^{-1}	1709.98	560.70	1149.28	67.21	67.68	0.011682
	1683.24	558.50	1124.74	66.82		
	1697.55	526.07	1171.48	69.01		
2.0 mg L^{-1}	2803.54	509.40	2294.14	81.83	81.53	0.023039
	2843.19	561.81	2281.38	80.24		
	2838.96	495.97	2342.99	82.53		
4.0 mg L^{-1}	5298.34	474.73	4823.61	91.04	91.67	0.011935
	5283.75	367.22	4916.53	93.05		
	5289.99	479.80	4810.19	90.93		
6.0 mg L^{-1}	7823.98	680.69	7143.29	91.30	91.54	0.008709
	7849.88	720.62	7129.26	90.82		
	7820.02	585.72	7234.30	92.51		
8.0 mg L^{-1}	9453.02	818.63	8634.39	91.34	92.11	0.008069
	9451.23	751.37	8699.86	92.05		
	9467.43	667.45	8799.98	92.95		

5. Aquaculture water samples

MPs concentrations	F_0	F_X	ΔF_X	RE_X (%)	Average RE (%)	STDEV
1.0 mg L^{-1}	1599.01	568.93	1030.08	64.42	64.75	0.009491
	1566.28	535.35	1030.93	65.82		
	1587.36	571.29	1016.07	64.01		
2.0 mg L^{-1}	3024.56	564.38	2460.18	81.34	81.41	0.014614
	3036.90	519.01	2517.89	82.91		
	3001.82	600.66	2401.16	79.99		
4.0 mg L^{-1}	5411.76	539.01	4872.75	90.04	91.28	0.010739
	5405.87	438.42	4967.45	91.89		
	5441.42	440.21	5001.21	91.91		
6.0 mg L^{-1}	8096.53	604.81	7491.72	92.53	90.90	0.014205
	8089.49	791.96	7297.53	90.21		
	8099.64	814.01	7285.63	89.95		
8.0 mg L^{-1}	9400.95	928.81	8472.14	90.12	90.38	0.011424
	9429.76	789.27	8640.49	91.63		
	9415.65	999.00	8416.65	89.39		

6. Inner-city moats samples

MPs concentrations	F_0	F_X	ΔF_X	RE_X (%)	Average RE (%)	STDEV
1.0 mg L^{-1}	1756.98	722.12	1034.86	58.90	59.57	0.014008
	1760.31	683.35	1076.96	61.18		
	1743.60	721.33	1022.27	58.63		
2.0 mg L^{-1}	2902.76	724.53	2178.23	75.04	75.53	0.013286
	2919.35	744.14	2175.21	74.51		
	2959.90	679.89	2280.01	77.03		
4.0 mg L^{-1}	5310.99	695.21	4615.78	86.91	88.00	0.011270
	5325.83	643.36	4682.47	87.92		
	5349.11	579.84	4769.27	89.16		
6.0 mg L^{-1}	7711.74	1085.04	6626.70	85.93	86.72	0.010489
	7739.21	1058.72	6680.49	86.32		
	7792.42	942.10	6850.32	87.91		
8.0 mg L^{-1}	9398.21	1322.33	8075.88	85.93	87.05	0.010166
	9372.58	1188.44	8184.14	87.32		

	9389.74	1135.22	8254.52	87.91		
